# A Comprehensive Narrative Review of Nanomaterial Applications in Restorative Dentistry: Demineralization Inhibition and Remineralization Applications (Part I)

**DOI:** 10.7759/cureus.58544

**Published:** 2024-04-18

**Authors:** Sahar M Elmarsafy

**Affiliations:** 1 Department of Restorative Dentistry, Faculty of Dental Medicine, Umm Al-Qura University, Makkah, SAU; 2 Department of Conservative Dentistry, Faculty of Dental Medicine for Girls, Al-Azhar University, Cario, EGY

**Keywords:** nanodentistry, dental restorations, composite resin, remineralization, demineralization, anti-caries, antibacterial, nanoparticles, nanotechnology

## Abstract

Nanotechnology is extensively employed in various aspects of dentistry, including restorative dentistry, because of its substantial improvement and promising potential in the clinical efficacy of restorative materials and procedures. The main purpose of this review is to explore the different uses of nanomaterials in restorative dentistry. The review is divided into two parts: the current review (Part 1) focuses on the prevention of demineralization and promotion of remineralization, while the upcoming review (Part 2) will discuss the reinforcement of restorative materials and their therapeutic applications. Nanofillers are added to dental materials to boost their antibacterial, anticaries, and demineralization inhibitory capabilities. Additionally, they improve remineralization and enhance both mechanical properties and therapeutic features. The nanoparticles (NPs) used to increase antibacterial and remineralization inhibitions can be classified into two main groups: inorganic and organic NPs. Examples of inorganic NPs include silver, zinc oxide, titanium oxide, and gold. Examples of organic NPs include silica, quaternary ammonium salt monomers, and chitosan NPs. Furthermore, the nanofillers utilized to enhance the process of remineralization include various types such as metals, nano-hydroxyapatite, nano-amorphous calcium phosphate (ACP), dicalcium phosphate NPs, casein phosphopeptide-ACP (CPP-ACP), and calcium fluoride NPs. These uses underscore the potential applications of NPs in restorative dentistry, although there are still some limitations to address.

## Introduction and background

"There's Plenty of Room at the Bottom: An Invitation to Enter a New Field of Physics" was a lecture delivered by physicist Richard Feynman on the idea of nanotechnology during a meeting of the American Physical Society held at Caltech on December 29, 1959. A nanometer is equivalent to one billionth of a meter in length. At this scale, particles exhibit distinct properties that diverge significantly from those observed in the same material at greater scales. As a result of this notion, lighter but stronger materials with improved physical, chemical, and biological properties have been developed. Since then, nanotechnology has made significant advances in various disciplines of science and dentistry [1].

The term "nano-" originates from the Greek word "νανος," which means "dwarf." It is typically used in conjunction with a noun to produce words like nanometer, nanotechnology, and nanorobot. In science, “nano-” denotes one billionth of a meter or a factor of 10−9. Because the human brain has difficulty conceptualizing such a tiny number, an analogy has been created to understand the nanoscale, which ranges from 1 nanometer (nm) to 100 nanometers. A substance with a size smaller than 100 nm in one dimension is commonly known as a nanomaterial, and this classification is determined by its structure. Another deeper definition takes into account not only the structure of the nanomaterial but also its features, namely, the unique features that are exclusive to its small size [1,2].

Nanotechnology is widely utilized throughout several areas of dentistry, particularly in restorative dentistry, endodontics, biomineralization, prosthodontics, periodontics, bone regeneration, implant odontology, oral cancer diagnosis, drug delivery, and nano-tissue engineering. The antibacterial characteristics, mechanical reinforcement nanostructure, and therapeutic approaches are the key factors contributing to the various benefits of nanotechnology [2].

Despite the long history of the production and development of various types of restorative materials for more than 100 years, including amalgam, gold, silicate cement, glass ionomer, resin composite, and ceramics, they have always suffered from some drawbacks in their performance inside the oral cavity. Conversely, the emergence of the field of nanotechnology is considered a great opportunity to address these drawbacks.

The application of nanotechnology in restorative dentistry is very influential due because of its significant enhancement in the clinical performance of restorative materials. Nanofillers are incorporated into dental materials to improve their antibacterial, anticaries, and demineralization inhibition properties. Additionally, they enhance remineralization, improve mechanical properties, and promote therapeutic approaches. The advent of the digital era and growing expectations of patients for both beauty and functionality pose important challenges for the scientific investigation of engineered nanomaterials [3].

Therefore, the primary objective of this narrative review is to explore the various applications of nanomaterials in restorative dentistry by critically analyzing selected relevant literature sources. The review was divided into two parts: the current review (Part 1) focuses on demineralization inhibition and remineralization applications, while the upcoming review (Part 2) will cover restorative reinforcement and therapeutic applications.

## Review

Nanomaterial fabrication and properties

Nanomaterials, often also called nanostructures, are classified based on grain structure and sizes into four categories. Zero dimensional (0D) refers to objects that have all dimensions in the nano-range, such as nanoparticles (NPs), quantum dots, and nanodots. The term one-dimensional (1D) refers to a nanostructure with one dimension in the nano-range, such as nanowires, nanorods, and nanotubes. Two-dimensional (2D) refers to nanostructures that have two dimensions outside the nanoscale range, such as coatings and thin-film multilayers. Three-dimensional (3D) refers to nanostructures, such as bulk nanostructures, that extend beyond the nanoscale range in all three dimensions [2].

Engineered NPs are intentionally made to have dimensions at the nanoscale, whereas incidental NPs are inadvertently formed as a result of combustion processes, industrial manufacturing, and other human activities. NPs exist in nature as various vital processes in organisms occur at the nanoscale. The human body utilizes natural nanoscale substances, such as proteins and other molecules, to regulate its diverse systems and activities [4].

NPs are unique in nature, and the scientific rationale for this distinction is attributed to numerous factors. One of these factors is the dominance of electromagnetic forces; between two protons, the electromagnetic force is 10^36 times stronger than the gravitational force. Gravitational forces diminish at the nanoscale, whereas electromagnetic forces become dominant. Another important factor explaining the behavior of nanomaterials is quantum effects; quantum, meaning "amount" in Latin, refers to the smallest discrete unit of any physical attribute, including matter or energy. Materials can display new properties that the same substances do not display at the micro- or macroscales, such as electrical conductivity, flexibility, increased strength, different colors, and stronger reactivity. Additionally, the surface-to-volume ratio plays a significant role in the nature of nanomaterials. A substance's chemical reactivity increases when its surface area-to-volume ratio increases as a given mass of material in the form of NPs will be significantly more reactive than the same mass of material composed of larger particles because growth and catalytic chemical reactions occur on the surfaces. Moreover, random molecular motions can significantly affect the way particles behave at the nanoscale because they can occur on the same scale as the particles' sizes [5].

The materials exhibit distinct characteristics when observed at the nanoscale. For instance, carbon in graphite form (like pencil lead), which is soft and malleable, displays enhanced strength at the nanoscale compared to steel and is six times lighter. Zinc oxide (ZnO), typically white and opaque, undergoes a change at the nanoscale, displaying as transparent. Additionally, aluminum, when reduced to the nanoscale, exhibits the ability to combust and is utilized as rocket fuel. Nanoscale materials offer numerous advantages and exhibit enhanced characteristics in comparison to their larger-scale counterparts. These advantages include improved strength, reduced weight, greater control over the light spectrum (which can be used to intensify light, direct it in a specific direction, or even turn it into different colors), and more chemical reactivity [1].

The primary methods employed to produce nanostructures are categorized as either bottom-up manufacturing or top-down manufacturing. Bottom-up manufacturing, also known as molecular nanotechnology, is a process that involves the construction of structures at the atomic or molecular level, resembling the method of bricklaying (e.g., plasma etching and chemical vapor deposition). The process of top-down manufacturing involves starting with substantial bulk material and subsequently employing processes such as etching, milling, or machining to selectively remove material and create a nanostructure [6].

Classifications of nanomaterials in restorative dentistry

Articles categorize and classify nanomaterials used in different disciplines of dentistry, mainly based on their shape, composition, and function [5-10].

The following classification consists of three subcategories of nanomaterials with different shapes: NPs, nanotubes, and nanoplatelets. Nanomaterials constructed from NPs can be classified as either conventional or non-conventional. Traditional NPs contain metal and metal oxide NPs, which have been thoroughly investigated for many years. The most recent fillers for advanced dental materials comprise non-traditional NPs (e.g., nanodiamonds (NDs), nanoshells, and quantum dots) that are easily adapted for different uses [5].

Conventional NPs

Metallic NPs are produced by utilizing the procedure of decreasing bigger particles into smaller ones and subsequently applying the resulting NPs onto a surface using a technique called sputtering. Examples of such NPs include silver NPs (Ag NPs), gold NPs (Au NPs), and copper NPs (Cu NPs). It is noteworthy that metallic NPs frequently exhibit antibacterial properties. Metallic particles in their oxide form exhibit greater stability compared to metallic particles alone. Metal oxide NPs, such as ZnO NPs (ZnO NPs), titanium dioxide NPs (TiO_2_ NPs), zirconium dioxide NPs (ZrO2 NPs), aluminum oxide NPs (Al2O3 NPs), and silicon dioxide NPs (SiO2 NPs), have been the focus of many recent studies exploring their potential as antibacterial agents [7].

Unconventional NPs

Nanodiamonds (NDs) are minimal diamonds having a diameter of less than 100 nm, and they are a great filler option because of their great surface and chemical nature in dental nanocomposite fabrication. Another type of unconventional NP is the quantum dot. Nanoshells consist of a thin metal shell enclosing a dielectric core, and these nanoshells have several therapeutic uses in dentistry. When exposed to infrared light, the metal coatings on nanoshells generate high heat, which can be utilized to destroy oral cancer cells. Other unconventional types include quaternary ammonium methacrylate (QAM), quaternary ammonium polyethyleneimine (QPEI), and amorphous calcium phosphate (ACP) NPs [5,7].

Nanomaterials Based on Carbon Nanotubes (CNTs)

CNTs have outstanding electrical and mechanical features. Single-walled CNTs (SWCNTs) are graphene-coated cylinders that attract interest among researchers. Dental resins that incorporate single-walled CNTs (SWCNTs) as fillers have shown exceptional flexural strength and have achieved excellent outcomes. Clay can be used to produce nanomaterials. Halloysite nanotubes (HNTs) are naturally occurring tubular clay nanomaterials constructed of rolled sheets of aluminosilicate kaolin and may serve as nano-drug delivery agents and dental fillings. HNTs are useful fillers in the production of dental composites because of their high strength, elastic modulus, and natural milky white color, in addition to their potential to be loaded with antibacterial drugs. Graphene can be used as nanoplatelet-based nanomaterials, such as nanosheets or flakes. Graphene oxide nanoplatelets have unique characteristics that render them exceptionally ideal for use in dental applications. Graphene is a material with unique properties made of carbon atoms arranged in a hexagonal honeycomb lattice called graphene oxide nanoplatelets. Graphene has been used in dental nanocomposites to increase the mechanical properties of the composite and act as an antibacterial agent as well as a filler [8].

Another classification and definition system for nanomaterials is provided, principally by their structures into metallic and nonmetallic categories [9].

Metallic Nanomaterials

There are various examples of metallic nanomaterials such as Ag, gold (Au), copper (Cu), zinc (Zn), and titanium (Ti). Ag NPs have been successfully utilized in the treatment of dental decay; they usually induce oxidative stress to demonstrate their antibacterial action. However, they fail to demonstrate any remineralization ability, just antimicrobial activity. Furthermore, the high cell toxicity and staining effects of many metallic NPs restrict their clinical use. Researchers are progressively looking towards nonmetallic NPs because of these drawbacks with metallic nanomaterials [7,9].

Nonmetallic Nanomaterials

Nonmetallic nanomaterials are devoid of metallic elements and have been shown to have remineralizing (biomimetic) and/or antibacterial characteristics in the literature. Furthermore, nonmetallic NPs show better biocompatibility than metallic nanomaterials, and their addition to dental materials does not substantially change the materials' color or method of use. Organic nanomaterials are the most widely used kind of nonmetallic nanomaterial used for caries treatment. Nonmetallic NPs can be added to toothpaste, dental adhesives, topical treatments, dental sealants, candies, and medications. Nonetheless, regarding the study of nonmetallic NPs' performance, few articles are clinical research, and the majority are in vitro investigations. Nonmetallic nanomaterials are classified into biological organic, synthetic organic, carbon-based, and selenium nanomaterials [10].

Nanomaterials used in restorative dentistry are also classified according to their function, as nanofillers are added to dental materials to enhance their antibacterial, anticaries, and demineralization inhibition properties, enhance remineralization, and improve mechanical properties, as well as for therapeutic approaches [10].

Overview of applications of nanomaterials in restorative dentistry

Nanotechnology is applied in nearly all fields of dentistry, including restorative dentistry, as it is an opportunity for a huge improvement in the restorative materials' clinical performance, mainly composite resins, dental adhesives, and glass ionomer materials. Aiming to enhance their strength and aesthetics, improving their therapeutic properties such as bioactivity and antibacterial effect to control, and diminish the bacterial biofilm on either the tooth surface or at the tooth-restoration interface [7]. Nowadays, nanocomposites, nano-filled adhesives, nano-ionomers, and even nano-amalgam restorative materials are introduced into the dental market.

Nanocomposites

Nanocomposites are either nanofilled or nanohybrid. Nanofilled composites contain a combination of nano-sized particles (nanomers) (5-75 nm) and agglomerated nanosized particles of 1.3 μm, defined as “nanoclusters.” To guarantee that the silane penetrates the cluster interstices, the agglomerated porous clusters were slightly calcined and infiltrated with a dilute silane coupling agent. A second, undiluted silane coupling agent was then mixed with the "nanoclusters" before they were incorporated into the resin matrix. However, nanohybrid composites combine nanometer-sized particles (20-60 nm) with more conventional filler technology (micron-sized filler particles of 0.1-2.5 μm) [8].

Recent studies have demonstrated that the incorporation of NPs into a resin matrix leads to improvements in numerous properties such as higher filler loading, which leads to improvement in mechanical properties (toughness, stiffness, wear and abrasion resistance, hardness, flexural strength, and inhibits crack formation and propagation). Nanocomposites also lead to a decrease in the quantity of resin present, resulting in a notable reduction in polymerization shrinkage, considering that polymerization shrinkage exhibits variability based on the composite's chemical structure as well as the manufacturing process. Moreover, nanocomposite restorative materials that incorporate Ag NPs have been found to have antibacterial properties [10].

Moreover, nanocomposites are also characterized by superior aesthetics. Nanofilled composites appear highly translucent, which is explained by the smaller size of the NPs than the wavelength of visible light (0.4-0.8 micrometers). Therefore, absorption does not occur, and light shines through without refractions. Additionally, nanoclusters break into individual primary particles resulting in surface defects that are smaller than the wavelength of light, retaining its polish for longer. The nanocluster is designed to fracture or break off rather than "plucked out," so the surface remains smooth and glossed. The smaller filler size will lead to a higher depth of cure and degree of conversion of the nanocomposites, and nanocomposites have good handling properties if the viscosity is controlled. Moreover, the particles have a spheroidal shape, which provides smooth and rounded edges that help in uniform stress distribution through the composite. This phenomenon is called the "roller-bearing effect" [11].

There is high evidence about the effectiveness of using nanofillers to improve the mechanical properties of composite resin. Ghahremani et al. [12] assessed a color-modified heat-cure resin with increased tensile and impact strength increased by titanium oxide (TiO_2_) NPs [12]. They incorporated TiO_2_ nanofillers into a triplex heat-cure resin and thoroughly mixed them. They then discovered that the strength of the tested group was significantly higher than the control group, particularly 7 MPa higher. The study revealed that adding 1% by weight of TiO_2_ to color-modified acrylic resin resulted in an increase in tensile and impact strength. However, the research did not investigate the possible adverse effects of this procedure on the restorative resin [12].

In the same context, Wang et al. [13] combined wrinkled mesoporous silica (WMS) with both unimodal and bimodal particles, and the resin matrix used in the mixture was based on bisphenol A-glycidyl methacrylate (BisGmA)/TEGDMA. Resin composites were produced using bimodal WMS fillers, specifically WMS-Si90 or WMS-Si190. The research revealed that the bimodal filler-blended WMS had superior biomechanical characteristics compared to its single-modal filler [13].

Moreover, the positive effect of adding different types of NPs to resin composite on its antibacterial, anticaries, and remineralization properties has been strongly proved through many studies [14-16]. Xiao et al. [14] constructed a bioactive multifunctional composite (BMC) by combining nanofillers of ACP (NACP), 2-methacryloyloxyethyl phosphorylcholine (MPC), dimethylaminohexadecyl methacrylate (DMAHDM), and Ag NPs). They also examined the impact of adding poly(amidoamine) (PAMAM) to the BMC. The research mainly examined root decay and showed remarkable remineralization of the root dentin, consequently producing a bioactive multifunctional resin composite for cervical restoratives. In addition to remineralization, the generated BMC exhibited protein-repellent properties and possessed antimicrobial characteristics [14].

Furthermore, in research conducted by Al-Eisa [15], ZnO NPs were utilized and integrated into a resin composite to assess its effectiveness as an antibacterial agent. The researcher conducted his experiments on agar with varying percentages, specifically 5%, 7%, and 10%. Among all identified types of bacteria exhibiting activity, Streptococcus mutans and Pseudomonas were found to be the most susceptible bacterial isolates, respectively. These data show that ZnO NPs have the potential to be highly effective in preventing secondary caries [15].

Moreover, Hubner [16] aimed to develop an antibacterial restoration defense against recurrent caries. He added octenidine dihydrochloride (OCT) to drug-eluting mesoporous silica nanomaterials, which were created using a bottom-up synthesis process. The added component is considered biocompatible, and no microbial resistance has been reported.

Given the numerous advantages of utilizing materials with nanofillers, it is essential to consider the challenges facing the development of nanocomposites. These challenges include avoiding particle agglomeration by providing effective dispersion and achieving a balance between high filler loading benefits and preserving adequate handling properties and low manufacturing costs. Moreover, the toxicity of NPs remains a significant challenge that requires further investigation and research [17]. 

Nanofilled Adhesives

Over the past decade, there has been an increase in interest in research on adhesive dentistry about strengthening the attachment between restoration materials and teeth. Nanotechnology aids various aspects of these improvements. Among the benefits of applying nanotechnology to adhesive systems are improved bond strength, mechanical properties, and elastic modulus of the adhesive layer. It also improves the distribution of stresses induced by resin composite (acting as an elastic buffer or shock absorber), leading to increased bond strength. The addition of NPs with specific properties, such as antibacterial or therapeutic NPs, allows for the development of bioactive adhesives that could reduce residual or invaded bacteria via microleakage. Some nano-filled adhesives may also promote remineralization of the demineralized/etched collagen beneath the hybrid layer [17].

Another exciting development in adhesive dentistry is the idea of self-healing adhesives. The idea of a self-healing polymer is based on the encapsulation of catalysts and monomers and then reinforcing them into the polymer. These capsules burst when the fractures are triggered, allowing healing monomers to fill the gap created by the crack and polymerize it. There is a critical need for a nanoencapsulating agent to enable therapeutic agents used in dental adhesives to permeate the extremely tiny submicron voids formed during acid etching. Research has reported on the use of polyurethane nanocapsules loaded with TEGDMA as a primary ingredient in self-healing bonding adhesive [17].

Yue et al. [18] developed a self-healing adhesive that has the ability to prevent the growth of microorganisms and stimulate remineralization by investigating the impact of including microcapsules, namely, DMAHDM, and NACPs, which had not been done before. The fracture toughness, Kic, and crack-healing efficiency were determined using the single-edge V-notched beam method. Yue et al. [18] realized three outcomes using the recently created adhesive resin: independent cracking repair, antimicrobial capacity, and remineralization activity by employing calcium phosphate (CaP) nanofillers.

Another innovative study done by Xie et al. [19] proposed a CaP ion-charging, protein-repellent, and antibacterial adhesive. A protein-repellent and antibacterial NACP-rechargeable adhesive was developed using 2-MPC and DMAHDM to fight biofilms and caries. The bioactive adhesive was protein-repellent and significantly inhibited bacterial adherence [19]. Conversely, the incorporation of nanofillers in adhesive systems suffers from problems that must be overcome as, to avoid the main particles of the adhesive solution from aggregating together forming "filler clusters" and becoming too large to infiltrate the interfibrillar spaces, it is important to ensure they are adequately stabilized during storage and/or application. These clusters might function as imperfections that lead to bond strength decrease and fractures. They may even prevent resin monomers from penetrating the spaces between collagen fibers, leading to voids and defects in the adhesive and hybrid layer. Moreover, consideration must be given to the amount of nanofillers added, as this might increase the adhesive system's viscosity [20].

Nano-Glass Ionomer

Nanostructures can be classified based on their origin (natural or synthetic), dimensions (0D for nanostructures, 1D for nanorods, and 2D for thin films), or structural configuration (carbon-based, metal-based, polymeric-based, and inorganic-based nanostructures). The types of nanostructures added to glass ionomer cement can be divided into three main groups. Each of these nanostructures has its benefits, limitations, and clinical performance [20].

Nanotechnologies have been utilized in resin-modified glass ionomers producing nano-ionomers (e.g., Ketac Nano GI Primer, 3M ESPE, MN) by adding NPs (nanomers) and nanoclusters into fluoroaluminosilicate (FAS) glass. This leads to various benefits such as aesthetic improvement of the final restoration and polishability. The fluoride release property is still effective and rechargeable because of the high surface area of the NPs; however, several investigations have demonstrated that the amount of fluoride released over time with Ketac Nano GI Primer was lower compared to conventional GIC. The shear bond strength (SBS) of nano-ionomer is good compared to conventional GICs, while the Knoop hardness of Ketac Nano GI Primer was found to be lower than that of resin-modified glass ionomers. A new nanofilled resin-modified glass ionomer cement (RMGIC), known as the Equia system (GC Corp., Tokyo, Japan), has just been produced. The fillers consist of 40-nm-sized silica powder on average. Because the nanofillers tend to agglutinate in the resin matrix, a 35-40-μm-thick layer is formed to provide a seal and protection for the surface of the restoration, as well as the adhesive interface between the resin and the tooth structure [21].

In recent years, various novel studies have been conducted regarding the addition of NPs to glass ionomer restorative material for its improvement. One of these studies was conducted by Paiva et al. [22], who accomplished a research project to develop polyacid formulations using photoreduction to incorporate Ag NPs into a polyacrylate solution of conventional glass ionomer cement (GIC). The objective was to retain the antibacterial properties of the Ag-added GIC while also assessing its handling and mechanical characteristics compared to conventional glass ionomer cement. The improved restorative material was evaluated against S. mutans. The study observed that the addition of Ag NPs to GIC resulted in outstanding antibacterial efficacy. The repair functioned based on the process of diffusion, which involved the dissolution of Ag ions (via oxidation) from the cement matrix. This finding suggests that the addition of Ag NPs to GIC can effectively inhibit tooth decay and prevent the establishment of biofilms on their surface [22].

Moreover, according to Renné et al. [23], the ability of glass ionomer cement to release fluoride ions was enhanced by the inclusion of nanohydroxyapatite powder. In accordance with other research, hydroxyapatite (HA) NPs improve the biomechanical and antimicrobial properties of glass ionomer cement [24].

Nanofilled Dental Ceramics

Nano-ceramic composite and nano-optimized moldable ceramics are very promising products that apply nanotechnology in dental ceramics. Nanofillers are employed to improve the capacity to polish and decrease the amount of wear, while nanopigments alter the color of the restoration to match the adjacent tooth (chameleon effect). Additionally, nano-modifiers improve the stability (non-slump) of the material and prevent it from adhering to equipment.

In 2009, Yang et al. developed a novel nano-sized Al2O3-boron nitride (Al2O3-BN) covering for tetragonal zirconia polycrystals (3Y-TZP) nanofilled ceramic composites used in computer-aided design/computer-aided manufacturing (CAD/CAM) systems. The newly developed machinable ceramic composites exhibit excellent mechanical characteristics and are suitable for use in all types of ceramic dental restorations [25].

*Nano-Amalgam* 

Amalgam, a widely utilized dental filling material, is now facing restrictions in several countries as researchers continue to debate its use. The alloy is subject to criticism because of its mercury level, its susceptibility to corrosion, and its limited adaptability in the body's environment.

An outside-the-box idea was about using Ag-copper (Ag-Cu phase) nano-powder as an additive to improve the corrosion behavior of dental amalgams. The addition of TiO_2_ NPs to dental amalgam improved its corrosion behavior, mercury release, and antibacterial activity. Hence, for the usage in dentistry, an amalgam/TiO_2_ NPs nanocomposite containing 1% of TiO_2_ NPs could be considered a biocompatible and bioactive restorative material with superior properties [26].

Another study done by Khodaei et al. [27] involved the preparation of an amalgam/nano-HA composite. This composite was produced using various percentages of the weight of HA NPs and then analyzed using scanning electron microscopy (SEM). The findings indicated that augmenting the weight percentage of HA led to an enhancement in the corrosion resistance of the composite and a substantial reduction in the release of mercury ions. Additionally, the compressive strength of the composite increased by 18% (p < 0.05) when HA was increased up to 1%, but subsequently declined because of the agglomeration of the HA NPs [27].

Nanofillers applications in restorative dentistry to enhance antimicrobial, anticaries, and demineralization inhibition properties

One of the primary goals in restorative and preventive dentistry is combating and resisting plaque film and tooth decay, which includes several approaches and strategies. The applications of nanotechnology in dentistry have helped to achieve this goal. The NPs used for this goal are categorized into inorganic and organic NPs, including Ag, ZnO, TiO_2_, gold (Au), silica (Si), quaternary ammonium salt (QAS) monomers, and chitosan NPs [9].

The mechanism of the antibacterial activity of metals is not fully understood. However, evidence suggests that the interaction between positively charged metal particles and negatively charged membranes of bacteria has significance. Moreover, the explanations for the antibacterial activity of NPs could be among the following: destruction and malfunction of the cell membrane (ZnO, TiO_2_, Ag, Au, chitosan, fullerenes), disturbances of electron transport and energy production (Ag, fullerenes), production of reactive oxygen species (ROS) (TiO_2_), inactivation of cell enzymes (chitosan), and inhibition of DNA replication (Ag, chitosan) [28].

The utilization of emerging nanomaterials (ENMs) as carriers for traditional antibacterial agents such as chlorhexidine (the active component present in mouthwashes), allows the controlled release of chlorhexidine from these nanocomposites. Nevertheless, the effectiveness of chlorhexidine may be limited because of its short duration of action. Conversely, the incorporation of antibacterial NPs into resin-based composites has been shown to have a durable harmful effect on cariogenic bacteria while preserving the physical and mechanical characteristics of the dental material [28].

Metal NPs

Throughout history, several metals such as Ag, zinc, gold, titanium, and copper have been widely used as antibacterial agents. Metals and their related oxides have extensive application in a variety of dental material uses, whether as a standalone component or linked with others. Recently, there has been an increasing interest in replacing conventional antibacterial metal powders, which are usually in the micron-sized range, with their nanoscale alternatives [7].

The greater bactericidal efficacy of nanometals can be because of their small size and larger surface area, allowing them to interact directly with bacterial cell walls. Several factors may influence the selection of metallic NPs. The choice of antibacterial ENMs depends mainly on the specific dental application. For instance, Ag, Zn, and TiO_2_ NPs are often used as antibacterial coatings for dental materials or incorporated into resin-based composites [7].

Certain metallic NPs, such as Ag and Cu NPs, have demonstrated efficacy against a broad spectrum of bacterial strains, including S. mutans, Staphylococcus aureus, P. aeruginosa, Escherichia coli, Enterococcus faecalis, S. sobrinus, and S. epidermidis. Additionally, zinc magnesium oxide (ZnMgO) NPs exhibit highly specific antibacterial activity against Gram-positive bacteria. The bactericidal activity of metal ENMs can be influenced by their size and shape as research indicates that materials with a particle size smaller than 10 nm are most effective against bacteria. Moreover, triangular NPs exhibit superior bactericidal properties in comparison to NPs having a spherical or needle-like shape. The antibacterial efficacy of certain ENMs can be improved through the utilization of ultraviolet (UV) radiation, as demonstrated by the example of crystalline TiO_2_ NPs [7,28].

Green Synthesized Metallic NPs

The synthesis of NP formulations with herbal medicine has recently become an important advancement in dentistry as biological metallic NPs can be produced from plant extracts to overcome the restrictions of herbal remedies. The utilization of plant extracts in the production of environmentally friendly metallic NPs, such as Ag, Au, and Fe NPs, has demonstrated greater efficacy in the treatment of various orofacial diseases compared to conventional approaches. Antibacterial NP manufacturing processes are costly and can have harmful impacts on biological systems, the environment, and human health because of the inclusion of hazardous and toxic components. As a result, methods for producing green NPs have been established. This alternative utilizes biological systems, such as yeast, fungus, bacteria, and plant extracts, in place of hazardous compounds [29].

Thus, green plant-synthesized Ag NPs can serve as substitutes for chemically synthesized Ag NPs in the prevention and treatment of dental diseases and infections because of their antibacterial, antifouling, and remineralizing properties. The Ag NPs produced from the leaf extract of Justicia glauca exhibited strong antibacterial activity against S. mutans, S. aureus, and various other microbes, either on their own or when applied in combination with azithromycin and clarithromycin.

In a study conducted by Ahmed et al. [30], the researchers utilized extracts from Ficus bengalensis, Azadirachta indica (A. indica), and Salvadora persica (S. persica) plants to synthesize natural Ag NPs, which demonstrated antibacterial properties against L. acidophilus, L. lactis, and S. mutans. The aqueous extracts of three separate components of rice grains, specifically rice bran (RB), rice husk (RH), and rice germ (RG), were used to create Ag NPs, which exhibited antibacterial action against S. aureus, E. coli, S. mutans, and C. albicans [30].

Silver NPs (Ag NPs)

Ag-based NPs at very low concentrations (0.5-1.0%) are effective against the bacterial biofilm. Ag NPs can be added to adhesive systems and composite resins, providing widespread anticariogenic action to them [31]. Several controlled clinical trials were conducted to assess the efficacy of a novel anticaries drug called nanosilver fluoride (NSF), designed to arrest active dentine caries without staining the teeth [32].

The antibacterial mechanism of Ag NPs is explained by several points as the presence of proteoglycans within bacterial cells and on the membrane allows them to serve as binding sites for Ag NPs and Ag ions. Consequently, the Ag disrupts the bacterial cell membrane, while the Ag ions can also interact with sulfuryl groups during protein synthesis, thereby interfering with bacterial DNA replication and damaging it. This leads to cell death. Recently, the antibacterial activity of Ag-NPs has been related to the formation of free radicals, specifically bactericidal ROS. The free radicals generated by the Ag-NPs induce bacterial cell membrane damage. High quantities of ROS can induce oxidative stress in cells under specific circumstances. Oxidative stress not only induces damage to the cell membrane but also impairs proteins, DNA, and intracellular functions, including the respiratory system [33,34].

During the preparation of materials with Ag NPs, some factors should be considered. For instance, NPs larger than 50 nm cannot diffuse and penetrate the biofilm, and the process of producing and dispersing Ag NPs is difficult because of the tendency of the particles to clump together. Concentrations above 0.02% negatively impact the mechanical properties as a result of increased aggregation, and higher concentrations also compromise the degree of conversion of the light-cured restorative material [17].

Not all dimensions of silver NPs exhibit equivalent antibacterial efficacy. Ginjupalli et al. [34] demonstrated that silver NPs with a size range of 80-100 nm are more effective in providing antimicrobial effects to irreversible hydrocolloid impression material compared to smaller particle sizes. The primary mechanism behind the antibacterial activity of Ag NPs is the release of silver ions [34].

ŁSokołowski et al. [35] assessed experimental resin composites modified with nanogold and nanosilver, which display reduced light transmission and have an opaque appearance. The microhardness of all experimental composites was greater than that of nonmodified resin composites, and the diametral tensile strength of the experimental composites was similar to that of the control group [35]. 

Furthermore, Ramzan et al. [36] conducted a study to synthesize silver NPs (Ag NPs) using extracts from wild ginger and assess the antibacterial effectiveness of these Ag NPs against multidrug-resistant (MDR) strains of S. aureus, S. mutans, and E. faecalis. The Ag NPs produced through biosynthesis demonstrated full antibacterial efficacy against the multidrug-resistant bacterial strains that were tested. Considering that there was evidence that an increase in the concentration of nanosilver caused an increase in the antibacterial properties of composite resin [37].

Silver NPs applied among different restorative materials in composite resins, cements, and adhesives showed very promising performance. Thus, a unique nanocomposite NACP-nanosilver (NACP-NAg) evolved. This nanocomposite has strong mechanical characteristics and powerful antibacterial capabilities. Moreover, it significantly reduces the survivability of biofilms and the formation of lactic acid. Thus, the NACP-NAg nanocomposites reveal great potential for dental restorations because of their ability to promote remineralization and exhibit antibacterial properties.

In 2012, an innovative approach was introduced for the first time. It involved adding two substances: NACP, a remineralizing agent, and silver NPs (Ag NPs), an antibacterial agent, to dentin adhesives and primer. The presence of Ag NPs and NACP in bonding chemicals significantly decreased the viability and generation of acids of dental plaque and biofilm but preserved the strength of the dentin bond. Wang et al. [38] stated that 0.10% Ag NPs generated in situ may be suitable to provide the one-step self-etching adhesive with an extended antibacterial capability without compromising adhesive performance.

Additionally, Jia et al. [39] developed an orthodontic bonding agent that included NACP-polydopamine-Ag (NPA). The purpose of this adhesive was to address white spot lesions. All groups of fillers had a cell survival rate exceeding 70%, indicating excellent biocompatibility. Moreover, the orthodontic adhesive with 0.2 wt.% NPA fillers exhibited a SBS of 11.89 ± 1.27 MPa. Additionally, both the orthodontic bonding agent blocks and the extract exhibited strong antimicrobial activities, resulting in a considerable decrease in the number of colonies [39].

The main problems that exist in using Ag NPs in dental materials are the aesthetics as the conventional macro-sized metals influence the color of aesthetic restorative materials. Conversely, utilizing nanotechnology has the potential to solve this issue by embracing unique material characteristics. Size reduction implies enlarging the contact surface, which is essential for the efficacy of silver, while also preventing tooth discoloration by utilizing low quantities. The toxicity of silver NPs (Ag NPs) was observed to be strongly correlated with the presence of free silver ions that are discharged into the surrounding medium. Another issue arises about the ability of Ag NPs to traverse the blood-brain barrier via transsynaptic transport, leading to their accumulation in the brain [33].

Zinc Oxide NPs (ZnO NPs)

ZnO NPs exhibit antibacterial properties against various bacteria, such as S. mutans. The mechanism behind their action involves their capacity to interact with the cell membranes of different bacterial species. Zinc strongly binds to lipids and proteins, leading to alterations in osmotic balance and raised membrane permeability. Moreover, ZnO NPs increase oxidative stresses within the bacterial cell because of their ability to generate zinc concentration (Zn2+) and ROS (i.e., hydrogen peroxide (H_2_O_2_)), which can also inhibit the growth of planktonic bacteria [33,40]. Problems in the use of ZnO NPs are mainly related to aesthetics because the penetration of visible light is compromised by the opacity of the ZnO NPs, and the problem is its toxicity [17]. 

Literature reviews recorded useful applications of ZnO NPs in various dental products such as fissure sealants, adhesives, composite resins, and toothpaste. It was proven that the composite resin containing ZnO NPs exhibited a considerably greater antibacterial activity on S. mutans compared to the composite resin containing Ag NPs [9]. 

Choi et al. [41] in their study incorporated different quantities of ZnO NPs into an available pit and tooth fissure sealer called BeautiSealant (SHOFU, Japan) to create experimental materials. The amounts used were as follows: commercial control at 0%, while ZnO at 0.5, 1, 2, and 4 wt%. The pit and fissure sealants with ZnO NPs have shown antibacterial activity against S. mutans without any detrimental impact on the physicochemical and mechanical characteristics. Therefore, these sealants can serve as excellent materials for preventing secondary caries [41].

Moreover, Alfaawaz et al. [42] conducted an experiment wherein they developed an adhesive and examined how the addition of ZnO NPs at concentrations of 5-10 wt.% affects the mechanical properties of the adhesive. The addition of ZnO NPs at concentrations of 5 and 10 wt.% enhanced the adhesive's microtensile bond strength (μTBS). Nevertheless, a decrease in viscosity was noted for both adhesives reinforced with NPs [42]

According to a study conducted by Spencer et al. [43], ZnO was added into Fuji Ortho LC (Tokyo, Japan) to produce mixes containing 13% ZnO and 23.1% ZnO. The adjusted adhesive agent was subjected to incubation with S. mutans for 48 hours in a diffusion disc experiment, which was employed to quantify the areas of bacterial suppuration. Furthermore, brackets were attached to bovine primary incisors using the adjusted adhesive agents, and the SBS was assessed using a universal testing machine. By adding ZnO to Fuji Ortho LC, the compound gained antibacterial characteristics that became stronger as the concentration of ZnO increased while maintaining the SBS of the original compound [43].

Additionally, it has been discovered that the toothpaste, which includes 0.1% o-cymen-5-ol, 0.6% zinc chloride (ZnCl2), and 0.320%w/w sodium fluoride (NaF), can decrease the formation of early caries lesions by reducing the demineralization of sound human enamel. This effect is observed when comparing it to a control toothpaste that contains 1,450 ppm F [30].

Titanium Dioxide NPs (TiO_2_ NPs)

Integrating TiO_2_ NPs into a polymer can result in significant enhancements over multiple aspects such as improved antimicrobial capability, enhanced mechanical properties (including elastic modulus, toughness, stiffness, microhardness, and flexural strength), and provided bond strength values that were equivalent to or exceeding those of the controls without NPs [44]. 

The antibacterial mechanism of action of TiO_2_ NPs attributed to the particles undergoing photocatalysis when exposed to UV radiation, and this process generates ROS, primarily H_2_O_2_ and hydroxyl ions (OH-), which disrupt the osmotic equilibrium of bacteria. Additionally, these reactive species can interfere with phosphorylation, leading to oxidative cell death. Interestingly, TiO_2_ NPs may also exhibit antibacterial activity even without UV irradiation, although the exact mechanism behind this effect is unknown.

In their study, Alrahlah et al. [44], planned to enhance a commercially available restorative material made of polymethyl methacrylate (PMMA) by including TiO_2_ and ZrO2 NPs as fillers in varying proportions. The objective was to examine the resulting changes in the material's physicomechanical properties. The findings indicated that the addition of a small quantity (0.5 wt%) of TiO_2_ and ZrO2 NPs had a significant impact on improving the physical, mechanical, and morphological properties of PMMA interim restorations [44]. Other research stated that when TiO_2_ NPs were mixed with light cure orthodontic composite paste (Transbond XT) as nanofillers, its antibacterial effects were enhanced, and enamel demineralization was reduced without compromising the SBS when compared to conventional composites [45].

Furthermore, Welch et al. [46] described the addition of TiO_2_ NPs to dental adhesives with the goal of combining the bioactivity and on-demand bactericidal effects. In a simulated bodily fluid, it emerged that the photocatalytic activity of an adhesive containing TiO_2_ interfered with the bacterial acidity and beneficially affected tooth remineralization [46].

Despite its favorable properties, unfortunately, using TiO_2_ NPs in dental resins has not been very successful (limitations) because of the inconsistent agglomeration of TiO_2_ NPs, and extensive radiopacity if larger aggregates are formed. Furthermore, it was noted that TiO_2 _NPs caused a pro-inflammatory response in human gingival fibroblasts, with the degree of the response being dependent on the concentration of the NPs [40].

Gold NPs (AU NPs)

Gold NP-based materials offer several key benefits that enhance the performance and durability of the restoratives. First, they provide effective protection against the growth of microorganisms responsible for dental caries and provide long-term protection against secondary caries. Additionally, these materials exhibit chemical neutrality, which means that they do not react with dental materials. Furthermore, they remain active even when exposed to the light used for curing restorations. Furthermore, this does not result in the alteration of prosthetic restorations' color and enhances the bonding of composite materials to tooth tissues, significantly enhancing the longevity of dental restorations [47]. 

An interesting aspect of gold NPs is that they possess much smaller dimensions than dentinal tubules, which ensures that they can enter the tubules without much resistance. Besides gold NPs exhibiting a clear size-dependent trend, this deviation of the melting temperature from the bulk value becomes dramatic at a size around 5 nm in diameter, ultimately reaching well under 50% of the bulk melting point of gold [48].

A study in 2018 aimed to examine the antibacterial properties of a novel cavity disinfectant called NanoCare Plus Silver Gold® (NanoCare), which has bacteriostatic and antifungal properties compared to 0.2% chlorhexidine (CHX) gluconate disinfectant, and they found that the NanoCare cavity disinfectant showed significant antibacterial efficacy against S. mutans [47].

The inclusion of gold NPs in dental adhesives is very impressive as shown in the study by Dadkan et al. [48], and they added colloidal gold NPs with sizes of less than 20 nm to the dentin adhesive in various concentrations to achieve favorable antibacterial properties. It was shown that the addition of gold NPs at the concentration of 5X (i) increased the flexural strength of dentin tooth adhesive by 75%, (ii) increased the micro-SBS by 60%, and (iii) increased the tensile diameter strength of adhesive by 65% compared to the based adhesive. The results showed also that pure gold NPs have shown no toxicity for the growth of cells, and the incorporation of gold NPs has increased the cell viability in the base adhesive.

*Quaternary Ammonium Salt (QAS) Monomers and Quaternary Ammonium Polyethylenimine (QPEI)* *NPs*

An advantage of quaternary ammonium monomers is that they are copolymerized with other monomers and provide long-lasting antibacterial effects, and attempts have been made to modify resin composites using derivatives of these polymers [6]. In a study by Cheng et al. [49], the utilization of nano-Ag in combination with quaternary ammonium dimethacrylate in the Scotchbond Multi-Purpose Primer (3M, Germany) showed the most favorable results, with an enhanced antibacterial impact and reduced lactic acid production [49].

The antibacterial mechanism of QPEI involves binding to the cell membrane and inducing bacterial lysis by causing cytoplasmic leakage. This is achieved through the action of highly active polycationic agents, which facilitate the absorption of positively charged polymers onto the negatively charged cell surfaces of the bacteria. QPEI NPs are a promising new type of antibacterial NP that show great potential in reducing the recurrence of bacteria during the restoration of teeth [50-51].

The outcomes of Zaltsman et al. [52] research revealed that the inclusion of QPEI NPs in an orthodontic adhesive resulted in durable antibacterial effects against S. mutans, without compromising the adhesive strength to enamel, the extent of double bond conversion during polymerization, or the biocompatibility of the adhesive [52].

Chitosan NPs (Chitosan NPs)

The antibacterial effect of chitosan is related to the destruction and malfunction of the cell membrane, inactivation of cell enzymes, and inhibition of DNA replication. Chitosan is present in mouthwash and toothpaste, and it has been added to different restorative materials.

Ali et al. [53] assessed the antibacterial activity and hardness of microhybrid and flowable resin-based composites (RBCs) after modifying them with the novel antimicrobial ingredient chitosan. The findings showed that actinomyces viscous lawn growth was unaffected by control, experimental, flowable, and microhybrid RBCs. Compared to the control and other experimental flowable RBC groups, the Vickers hardness (VH) of the flowable RBCs containing 1% chitosan was significantly higher [53].

Nanofiller applications in restorative dentistry to enhance remineralization

Demineralization of the tooth structure refers to the dissolution of calcium and phosphate ions into the saliva and occurs because of an acid attack and results in the cavitation of the surface of the tooth. Demineralization is reversible through remineralization, which is mineral precipitation onto the tooth structure [54]. Remineralization is a significant progress in treating damaged enamel surfaces. The use of NPs in fissure sealants, fluorides, and toothpaste can help prevent tooth decay, a concept known as Nano-Prevention. However, additional investigation into the characteristics of these materials and the enduring dependability of these treatment approaches is needed [55]. The nanofillers that were used for remineralization purposes were: metal NPs, nano-HA, NACP, dicalcium phosphate NPs, CPP-ACP (CPP-ACP), and calcium fluoride NPs.

Metal NPs 

Toothpaste containing Zn-carbonate HA (Zn-CHA) NPs can help heal the surface of enamel by forming a protective biomimetic CHA layer, a protective coating rich in hydroxyapatite, which was revealed in a study by Lelli et al. [56]. In this study, which took eight weeks, two groups of 18- to 75-year-olds used toothpaste containing potassium nitrate/sodium fluoride (control group) and toothpaste containing Zn-CHA nanocrystals (testing group). Utilizing elementary analysis, X-ray diffraction (XRD), and infrared analysis, SEM can characterize teeth morphology and chemistry. The study found that Zn-CHA nanocrystals toothpaste remineralized enamel by depositing a hydroxyapatite-rich layer. However, nitrate potassium/sodium fluoride and nonspecified fluoride toothpastes were unable to modify the enamel surface [56].

A zinc fluoride (ZnF) combined solution demonstrated a much higher level of remineralization compared to fluoride (F) alone. This could be attributed to the ability of zinc in ZnF to maintain a higher level of porosity in the surface zone, which, in turn, facilitates a greater level of remineralization in the lesion body [28,57].

Prospective controlled clinical research by Santos et al. [32] examined the efficacy of NSF, a novel caries-arresting agent, applied annually to children. One hundred and thirty decaying deciduous teeth were randomly assigned to NSF (experimental agent) or water (control). One masked examiner diagnosed and treated teeth, and another calibrated examiner monitored caries arresting at seven days, five months, and 12 months. At seven days, 81% of NSF teeth had arrested caries, but none in the controls had. The NSF group had 72.7% arrested caries after five months, while the control group recorded 27.4%. At one year, 66.7% of NSF-treated lesions were healed, compared to 34.7% in the control group. The study's authors concluded that NSF successfully reduced the prevalence of dental cavities in children in communities with low incomes [32].

Furthermore, Mohammed et al. [58] examined the effect of Zn2+ on enamel demineralization in vitro under caries-simulating settings to clarify zinc's demineralization-reducing mechanism. Enamel blocks and synthetic HA were demineralized at pH 4.0 and 37°C in zinc-containing acidic solutions (0-3,565 ppm (Zn2+)). Ion release into the solution was measured using inductively coupled plasma-optical emission spectroscopy (ICP-OES). Attenuated total reflectance-Fourier transform infrared spectroscopy (ATR-FTIR) was used for assessing enamel blocks, while X-ray diffraction and neutron diffraction were used to analyze HA. This study reveals that zinc decreases enamel demineralization, and the zinc may form an α-hopeite-like phase on enamel surfaces at PO43: locations in the HA lattice under the examined conditions [58].

HA NPs

Although HA has excellent biocompatibility, it has not been widely used as a load-bearing material because of its poor mechanical properties. Additionally, the use of HA particles may restore the mineral content of carious dentin at the interface between resin and dentin. CaP is the main element found in teeth and bones, and it is the last stable product of the process of precipitation of calcium and phosphate ions in solutions. HA has a high affinity for protein, and it attaches to plaque bacteria and glycoproteins in the oral cavity, making it easier to remove by rinsing after brushing [59].

Da Silva et al. [60] studied the antibacterial and anti-inflammatory benefits of toothpaste with HA NPs (5-10%), xylitol (2-3%), and propolis (1-2%), with or without 1,500 ppm fluoride (F). The conventional toothpaste (1,500 ppm F) and experimental toothpaste with the greatest agent proportions seemed to decrease demineralization and improve remineralization [60]. 

One method to enhance the mechanical properties of HA NPs involves promoting the growth of crystals in a one-dimensional manner, which results in the formation of nanorods; this might give promising material [59].

Furthermore, Ai et al. [61] investigated a one-dimensional nanofibrous filler for resin composite, seeking antimicrobial and reinforcing effects. The hydrothermal synthesis of HA nanowires was achieved by utilizing calcium oleate as a precursor. To coat these nanowires, they were soaked in an aqueous polydopamine (PDA) and were then remedied with Ag NPs. These Ag NP-HA nanowires were made by thermo-curing with a 50/50 Bis-GMA mix. The modified HA-PDA-Ag wires strongly adhered to the Bis-GMA resin matrix and strengthened it. The combination showed excellent bactericidal activity without cytotoxicity, making it a perfect nanofiller [61]. 

Nano-Amorphous Calcium Phosphate NPs (NACP NPs)

HA is the final product of the transformation of ACP, the first precursor. ACP composites were developed and were found to release high concentrations of calcium (Ca) and phosphate (PO4) ions in water-based solutions, and the enamel lesions were successfully remineralized in laboratory experiments; however, its major drawback is that they were mechanically weak. NACPs were incorporated in resin composites to utilize the high surface area of these NPs, and large amounts of calcium (Ca) and phosphate (PO4) were released with a relatively small amount of NACP filler present, allowing for the addition of other fillers to help reinforce the resin [5].

NACP nanocomposite is characterized by various beneficial properties such as the release of calcium and phosphate ions similar to traditional CaP composites, an increase in flexural strength and elastic modulus for load-bearing restorations, and effective neutralization of a lactic acid challenge. Moreover, within biofilms and plaques, there exists a substantial supply of ions that can be discharged during acid attacks. This supply is considered "smart" as it significantly enhances the release of ions at a pH level of 4, which is the most critical point for inhibiting tooth decay [62].

One of the studies that revealed the positive effect of NACP NP applications was Akbarzade et al. [63], who synthesized and characterized nano-bioactive glass (nBG) and assessed its impact on enamel remineralization on pH-cycled, synthetically demineralized enamel surfaces using CPP-ACP including nBG (CPP-ACP-nBG). Researchers found that experimental groups had significantly greater microhardness than controls. SEM images showed HA in the all-tested groups [63].

Moreover, consequently, in the study conducted by Tao et al. [64], it was concluded that the adhesive, which contains NACPs, successfully remineralized dentin lesions in a biofilm model. Its potential to preserve the bond interface, prevent secondary caries, and extend the lifespan of restorations is encouraging [64].

Dicalcium Phosphate Anhydrous NPs (DCPA NPs)

DCPA NPs are a stable form of calcium phosphate. Attempts have been used to incorporate them into resin composites along with whiskers for reinforcement. Dara et al. [65] created composites of nano-DCPA (dicalcium phosphate anhydrous) whiskers that display excellent strength and the ability to release Ca and PO4 ions, which are used to fight tooth decay. They discovered that when nano-DCPA was combined with whiskers, it resulted in composites that released significant amounts of calcium (Ca) and phosphate (PO4), which enhanced the process of remineralization (through reprecipitating and forming HA inside tooth lesions and outside the tooth restoration interface while maintaining the strength of the resin composite) [65].

C*asein Phosphopeptide-Amorphous Calcium Phosphate Nanocomplex (CPP-ACP NPs)*

This protein nanotechnology involves the integration of specific phosphoproteins derived from bovine milk with the creation of NACPs. This innovative product aids in the process of remineralization and serves as a preventive measure against dental caries. CPP can deliver CaP and can also help the ACP bind with the dental enamel [33].

Numerous in vitro and in vivo studies have demonstrated the efficacy of various dental products such as varnish, adhesives, and mouth rinse, which contain CPP-ACP in repairing early white spot lesions [66-68]. As Fernando et al. [66] found in a laboratory and in a double-blinded randomized controlled, crossover design in situ clinical investigation, stannous fluoride (SnF2) and CPP-ACP produce a nanofilament coating on teeth and stimulate dental remineralization significantly. Sn (II) cross-linking CPP-ACP stabilizes complexes, enhancing transfer to the tooth surface and improving binding and ion absorption into the tooth mineral.

CPP and ACP combine to create nanoclusters, which results in a reservoir of calcium and phosphate. This reservoir helps sustain the high concentration of both substances in saliva. CPP-ACP can stabilize calcium and phosphate in a solution, which in turn aids in buffering the pH of plaque. Consequently, the concentrations of calcium and phosphate in plaque are increased. Hence, the concentration of calcium and phosphate continues to be high in the subsurface lesions, leading to remineralization. The mechanism of action of CPP-ACP against decay in teeth involves attaching to adhesin molecules on S. mutans, preventing their attachment to dental plaque and increasing the levels of calcium ions in the plaque, inhibiting plaque fermentation [69].

Fluoride NPs (F NPs)

Efforts have been made to develop restorative materials that contain fluoride as these materials release F ions that, when embedded in tooth enamel, could result in fluorapatite or F-enriched hydroxyapatite, which is less soluble than hydroxyapatite. Fluoride-releasing restoratives are promising in helping to control the problem of recurrent caries by promoting remineralization and inhibiting microbial growth and metabolism. The advantage of using fluoride in the form of NPs is that the large surface area of the NPs allows the use of a small amount of the filler while maintaining high fluoride release, and the fluoride-functionalized NPs can increase the formation of apatite, which can prevent demineralization and encourage remineralization [70].

Mitwalli et al. [71] developed a novel dental nanocomposite containing DMAHDM, 2-MPC, and NPs of calcium fluoride (nCaF2) for preventing recurrent caries via antibacterial, protein-repellent, and fluoride-releasing capabilities. This novel nanocomposite nCaF2+DMAHDM achieved strong antibacterial and ion-release capabilities, without compromising the mechanical properties. This bioactive nanocomposite is promising for reducing biofilm acid production, inhibiting recurrent caries, and increasing restoration longevity.

Health hazards in nanotechnology application

Despite the specific engineered NPs in dentistry having significant advantages that surpass their limitations, there are still scientific areas that require additional investigation, especially their clinical performance. Currently, there is a demand for focused research on the interactions between NPs and oral biofilms [20]. 

According to the literature, some NPs can remain in the body for longer than desired, making their safety a matter of extreme significance. Nanomaterials can pass through biological membranes, including the blood-brain barrier, and gain entry into cells, tissues, and organs that are often inaccessible to larger particles. Nanomaterials can enter the bloodstream by inhalation or ingestion, and certain types can even permeate the skin [72].

NPs exhibit a substantial surface-to-volume ratio, which enhances their level of reactivity. These characteristics, combined with their small size that enables NPs to penetrate biological barriers, make NPs potentially harmful to living organisms and toxic to cells. However, our understanding of the complex interactions between NPs and biological systems remains essentially inadequate. There is a general agreement that unbound NPs, particularly, may present serious health hazards. When it comes to composites, NPs are incorporated into a polymer matrix and held in place, which means that the potential harm from these particles appears to be minimal. However, these particles can be released and enter the digestive system by abrasive processes such as intraoral wear and finishing/polishing treatments [2].

## Conclusions

Nanomaterials have demonstrated favorable results for a variety of current and prospective dental applications, and researchers have utilized nanotechnology to improve the physical, mechanical, and therapeutic properties of existing materials and develop novel materials. Nanofillers are incorporated into dental restorative materials to augment their antibacterial, anticaries, and demineralization inhibitory characteristics as well as to improve remineralization, improve the material's mechanical properties, and promote therapeutic applications. The NPs that are used to enhance the antibacterial and remineralization inhibitions are categorized into inorganic and organic NPs, including silver, ZnO, TiO_2_, gold, silica, QAS monomers, and chitosan NPs. Moreover, the nanofillers that are used to promote remineralization include metals, nano-hydroxyapatite, NACP, dicalcium phosphate NPs, CPP-ACP, and calcium fluoride NPs. All of these applications are very promising and provide insights into the practical use of NPs in the field of restorative dentistry, although there are still some limitations that must be addressed in the future.
